# Supplementary material for: Beyond Participation: Evaluating the Role of Patients in Designing Oncology Clinical Trials
Source: Curr Oncol. 2023 Sep 8;30(9):8310–27. doi: 10.3390/curroncol30090603 (PMC10527717; doi:10.3390/curroncol30090603)
Supplement: Supplementary file 1 [file curroncol-30-00603-s001.zip › curroncol-2585382-supplementary.pdf]

Table S1. Search strategy used to identify relevant studies on the active engagement of patients in the design of oncology clinical trials.

Search term	Search number	Keywords/MeSH terms
Cancer	1	'neoplasm'/exp OR (Neoplasms) OR (Cancer) OR (Cancers) OR (Neoplasia) OR (Neoplasm) OR (Tumors) OR (Tumor) OR (Malignancy) OR (Malignancies) OR (Malignant Neoplasms) OR (Malignant Neoplasm) OR (Neoplasm Benign) OR (Cancer-related)
Research	2	(Clinical Trial) OR (Clinical research) OR (Research Design) OR (Health Research)
Patient Involvement		(Patient engagement) OR (Patient-centered) OR (Patient involvement) OR (Patient Perspective) OR (Caregiver Engagement) OR (Caregiver Perspective) OR (Patient participation) OR (Caregiver Participation) OR (Patient-led)
Aggregation of search terms	3	1 AND 2 AND 3
